# Efficacy of Delayed Therapy with Fosmanogepix (APX001) in a Murine Model of Candida auris Invasive Candidiasis

**DOI:** 10.1128/AAC.01120-19

**Published:** 2019-10-22

**Authors:** Nathan P. Wiederhold, Laura K. Najvar, Karen J. Shaw, Rosie Jaramillo, Hoja Patterson, Marcos Olivo, Gabriel Catano, Thomas F. Patterson

**Affiliations:** a University of Texas Health Science Center at San Antonio, San Antonio, Texas, USA; b South Texas Veterans Health Care Center, San Antonio, Texas, USA; c Amplyx Pharmaceuticals, Inc., San Diego, California, USA

**Keywords:** glycosylphosphatidylinositol anchor biosynthesis pathway, APX001, APX001A, *Candida auris*, experimental candidiasis, manogepix, fosmanogepix, Gwt1, fluconazole resistance, invasive candidiasis, murine model

## Abstract

The emerging pathogenic yeast Candida auris is associated with antifungal resistance and high mortality. The novel antifungal agent manogepix (APX001A) inhibits glycosylphosphatidylinositol-anchored protein maturation and has demonstrated activity against numerous pathogenic fungi, including C. auris. Our objective was to evaluate the *in vivo* efficacy of fosmanogepix, the *N*-phosphonooxymethyl prodrug (APX001), following delayed initiation of therapy in a murine model of C. auris invasive candidiasis. Neutropenic mice were intravenously infected with a fluconazole-resistant clinical isolate of C. auris. Twenty-four hours postinoculation, treatment began with vehicle control, fosmanogepix (104 and 130 mg/kg of body weight by intraperitoneal injection three times daily, or intraperitoneal 260 mg/kg twice daily), fluconazole (20 mg/kg by oral gavage once daily), or caspofungin (intraperitoneal 10 mg/kg once daily) and continued for 7 days. Fungal burden was assessed via colony count in the kidneys and brains on day 8 in the fungal burden arm and on day 21 as the mice became moribund in the survival arm. Significant improvements in survival were observed in each group administered fosmanogepix and caspofungin. Similarly, reductions in fungal burden were also observed in both the kidneys and brains of mice treated with the highest dose of fosmanogepix in the fungal burden arm and in each fosmanogepix group and with caspofungin in the survival arm. In contrast, no improvements in survival or reductions in fungal burden were observed in mice treated with fluconazole. These results demonstrate that fosmanogepix is effective *in vivo* against fluconazole-resistant C. auris even when therapy is delayed.

## Abstract (precis)

The emerging pathogenic yeast Candida auris is associated with antifungal resistance and high mortality. The novel antifungal agent manogepix (APX001A) inhibits glycosylphosphatidylinositol-anchored protein maturation and has demonstrated activity against numerous pathogenic fungi, including C. auris.

## INTRODUCTION

Candida auris has emerged as a significant clinical pathogen, which has quickly spread to multiple countries in several continents, with numerous institutional outbreaks of invasive disease and colonization having been reported in the literature (1–3). This emerging pathogen also poses challenges to infection control, as this species can be transmitted between persons in health care facilities (4, 5) and may persist on environmental surfaces for 1 to 2 weeks (6, 7), and quaternary ammonium compounds commonly used as disinfectants in health care facilities may be ineffective against this species (8, 9). Treatment options are limited against infections caused by this pathogen due to its propensity to form biofilms and high rates of antifungal resistance. Most isolates (∼90%) are resistant to fluconazole, and up to 50% may also have reduced susceptibility to other triazoles, specifically voriconazole (1, 3). Resistance to the echinocandins has also been reported (3, 10, 11), although this class of antifungals are still recommended for the treatment of invasive infections caused by C. auris (12). A small portion of isolates have also been reported to be resistant to all clinically available antifungal classes, including the polyene amphotericin B (13). Given the rapid spread of this emerging infection, the high mortality rates associated with invasive disease (1), and limited treatment options in the face of antifungal resistance, new therapeutic agents and treatment strategies are needed.

Manogepix (APX001A, formerly E1210; Amplyx Pharmaceuticals, Inc., San Diego, CA) is a novel antifungal agent that inhibits the inositol acyltransferase Gwt1 in the glycosylphosphatidylinositol (GPI) anchor biosynthesis pathway, thereby preventing GPI-anchored protein maturation (14). This agent is administered as the *N*-phosphonooxymethyl prodrug fosmanogepix (APX001, formerly E1211), which is rapidly converted to the active moiety following intravenous (i.v.) or oral administration. Studies have demonstrated that manogepix inhibits inositol acylation in fungi, including *Candida* and *Aspergillus* species, but not the human form of this enzyme (14). Thus, the potential for human toxicity is limited. Previous studies have demonstrated both *in vitro* and *in vivo* activity against various pathogenic yeasts, including Candida albicans, Candida glabrata, Cryptococcus neoformans, and Coccidioides posadasii, as well as filamentous fungi, including Aspergillus, Fusarium, and Scedosporium species (15–26), although it lacks *in vitro* activity against Candida krusei and some of the Mucorales (15, 16). Two recent studies have also reported *in vivo* efficacy against invasive disease caused by C. auris (27, 28). However, in both studies, therapy was started soon after intravenous inoculation (2 hours). Thus, it is unknown how effective fosmanogepix would be with delayed initiation of treatment. This may be of clinical importance given the challenges associated with the diagnosis of infections caused by this species (3, 29), which may delay therapy. The objective of this study was to evaluate the *in vivo* efficacy of fosmanogepix in an experimental model of invasive candidiasis caused by C. auris, where the start of therapy was delayed until 24 hours after inoculation.

## RESULTS

### *In vitro* susceptibility.

Manogepix demonstrated potent *in vitro* activity against C. auris isolates, with MICs ranging from ≤0.002 to 0.03 μg/ml and MIC_50_ and MIC_90_ values of 0.03 μg/ml and 0.125 μg/ml, respectively. The geometric mean (GM) MIC was 0.013 μg/ml. In contrast, the MIC range for fluconazole was 2 to >64 μg/ml, with MIC_50_ and MIC_90_ values each at >64 μg/ml. Caspofungin MIC values ranged from 0.06 to 0.5 μg/ml, and the MIC_50_ and MIC_90_ values were 0.25 μg/ml and 0.5 μg/ml, respectively. The MIC values for manogepix, fluconazole, and caspofungin against the isolate used in the infection model were 0.03 μg/ml, >64 μg/ml, and 0.25 μg/ml, respectively.

### Pharmacokinetics and dose tolerability.

The pharmacokinetic parameters determined in uninfected mice dosed with fosmanogepix in the preliminary pharmacokinetic/dose tolerability study are shown in Table 1. The overall exposures obtained after the last dose on day 7 (area under the concentration-time curve from 0 to infinite time [AUC_0-inf_]) ranged between 11.3 to 58.4 μg·h/ml. Since the different cohorts were dosed with fosmanogepix twice a day (BID) or three times a day (TID), total daily exposures ranged from approximately 34 to 175 μg·h/ml for the active component manogepix. Increases in manogepix exposure were greater than the proportional increases in the fosmanogepix doses administered. Manogepix, administered as the prodrug fosmanogepix, appeared to be well tolerated, with the exception of mice that received the 260 mg/kg of body weight TID dose, where 5 of the 21 mice were observed to be moribund prior to the study endpoint. Based on these results, the top dose of fosmanogepix used in the *in vivo* efficacy study was 260 mg/kg BID.

**TABLE 1:** Plasma pharmacokinetic parameters of manogepix in uninfected, neutropenic mice following 7 days of administration with the prodrug fosmanogepix

Pharmacokinetic parameter^*a*^	Values by fosmanogepix dose			
	78 mg/kg TID	130 mg/kg TID	260 mg/kg BID	260 mg/kg TID
*C*_max_ (μg/ml)	6.82	6.97	49.0	44.9
*T*_max_ (h)	0.5	1.0	0.5	0.5
Half-life (h)	0.9	1.1	1.7	2.0
AUC_0-last_ (μg·h/ml)	11.3	13.5	52.7	58.0
AUC_0-inf_ (μg·h/ml)	11.3	13.6	52.8	58.4
Calculated total daily exposures, AUC_0-inf_ (μg·h/ml)	33.9	40.8	105.6	175.2

a *C*_max_, maximum concentration of drug in serum; *T*_max_, time to maximum concentration of drug in serum; AUC_0-last_, area under the concentration-time curve from 0 h to last time point.

### Survival.

**FIG 1:**
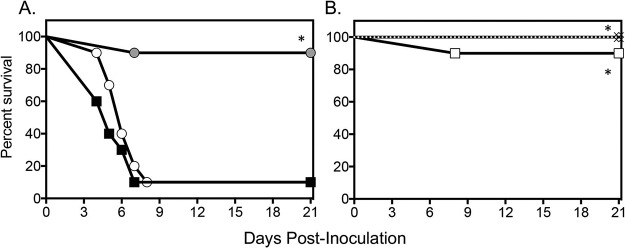
Survival curves in mice inoculated intravenously with C. auris and treated with vehicle control, fluconazole 20 mg/kg p.o. QD, or caspofungin 10 mg/kg i.p. QD (A) or fosmanogepix at doses of 104 mg/kg and 130 mg/kg i.p. TID or 260 mg/kg i.p. BID (B). Treatment started 1 day postinoculation and continued for 7 days. Mice were then followed after therapy stopped until day 21 postinoculation (total 14 days of no therapy). Black square, vehicle control; white circle, 20 mg/kg fluconazole; gray circle, 10 mg/kg caspofungin; white diamond, 104 mg/kg fosmanogepix; black X, 130 mg/kg fosmanogepix; white square, 260 mg/kg fosmanogepix. *n* = 10 mice in the vehicle control and each treatment group.

A survival advantage was observed in mice treated with fosmanogepix at clinically relevant doses (Fig. 1) (30, 31). At each dose level, median survival (>21 days for each group) and percent survival (range, 90% to 100%) were both significantly greater than the vehicle control (5 days and 10%, respectively; *P* < 0.0001 for each comparison). Survival was also significantly improved in mice that received a dose of caspofungin that results in exposures in mice that are 3- to 6-fold greater exposures than the standard dose used clinically (>21 days and 100%; *P* < 0.0001 versus vehicle control) (32–35). In contrast, there was no difference in median survival or percent survival compared with that of the control in fluconazole-treated mice (6 days and 10%). This finding is consistent with the *in vitro* fluconazole resistance observed for the clinical isolate used for infection.

### Fungal burden.

**FIG 2:**
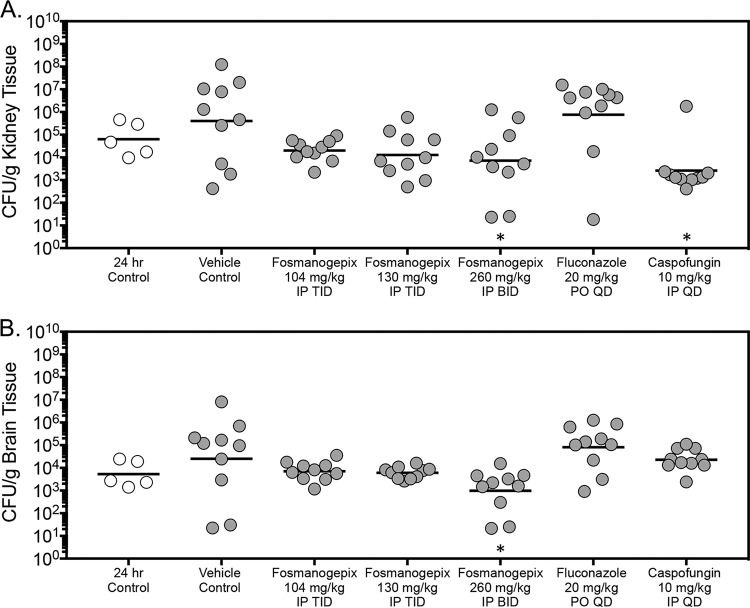
Kidney (A) and brain (B) fungal burden (CFU/g) in mice with invasive candidiasis secondary to C. auris in the fungal burden arm. CFUs were measured on day 8 postinoculation after 7 days of therapy. *, *P* < 0.05 versus control; *n* = 10 mice in the vehicle control and each treatment group; *n* = 5 mice in the 24-hour control group.

Following 7 days of therapy, kidney fungal burden was significantly reduced in mice treated with 260 mg/kg BID fosmanogepix (mean, 3.86 log_10_ CFU/g; *P* = 0.0273) or with 10 mg/kg intraperitoneal (i.p.) once a day (QD) caspofungin (3.41 log_10_ CFU/g; *P* = 0.0033) compared with that of the vehicle control (5.61 log_10_ CFU/g) (Fig. 2A). In contrast, while fungal burden was numerically reduced in mice treated with fosmanogepix at doses of 104 or 130 mg/kg TID (range, 4.11 to 4.30 log_10_ CFU/g), these differences were not significant. However, there was a trend toward significance in the 130 mg/kg TID fosmanogepix group compared with that of the vehicle control (*P* = 0.076). No reduction in fungal burden was observed in the kidneys of mice treated with fluconazole (5.88 log_10_ CFU/g). Similar results were also observed in the brain tissue in mice treated with fosmanogepix, as a significant reduction in CFU counts was observed in mice treated with the 260 mg/kg dose TID (2.99 log_10_ CFU/g) compared with that of the vehicle control (4.40 log_10_ CFU/g; *P* = 0.0088) (Fig. 2B). In contrast, no significant reductions in fungal burden were observed between the vehicle control group and mice treated with the lower doses of fosmanogepix (range, 3.78 to 3.85 log_10_ CFU/g), fluconazole (4.91 log_10_ CFU/g), or caspofungin (4.36 log_10_ CFU/g). Overall, the *in vivo* activity observed following 7 days of fosmanogepix therapy was static in nature since treatment with this investigational agent resulted in reductions in fungal burden of less than 1 log_10_ CFU/g compared with that measured just prior to the start of therapy (range, −0.50 to −0.94 log_10_ CFU/g) (Table 2). Treatment with a high (10 mg/kg) dose of caspofungin, which results in exposures 3 to 6 times that obtained in humans at the clinical dose did result in cidal activity within the kidneys (−1.39 log_10_ CFU/g) but not in the brain tissue (0.63 log_10_ CFU/g) (32–35).

**TABLE 2:** Changes in fungal CFU per gram of tissues for each treatment group compared with that prior to the start of therapy in both study arms

Treatment group	Log_10_ change^*a*^ (95% CI) in fungal CFU/g by study arm and tissue type			
	Fungal burden		Survival	
	Kidney	Brain	Kidney	Brain
Vehicle control	0.80 (−1.46 to 3.07)	0.68 (−0.92 to 2.28)	1.85 (−0.10 to 3.81)	1.80 (−0.11 to 3.72)
Fosmanogepix, 104 mg/kg TID	−0.50 (−2.76 to 1.76)	0.13 (−1.47 to 1.73)	−1.52 (−3.47 to 0.44)	−1.46 (−3.37 to 0.46)
Fosmanogepix, 130 mg/kg TID	0.69 (−2.95 to 1.57)	0.06 (−1.54 to 1.66)	−1.50 (−3.46 to 0.46)	−1.29 (−3.20 to 0.63)
Fosmanogepix, 260 mg/kg BID	−0.94 (−3.20 to −1.32)	−0.73 (−2.33 to 0.87)	−1.57 (−3.53 to 0.38)	−1.44 (−2.29 to 0.51)
Fluconazole, 20 mg/kg QD	1.08 (−1.18 to 3.34)	1.19 (−0.42 to 2.78)	2.11 (0.15 to 4.07)	2.05 (0.13 to 3.96)
Caspofungin, 10 mg/kg QD	−1.39 (−3.65 to 0.87)	0.63 (−0.96 to 2.23)	−2.78 (−4.74 to −0.82)	−1.86 (−3.78 to 0.05)

a All values are compared with that of the 24-h control.

**FIG 3:**
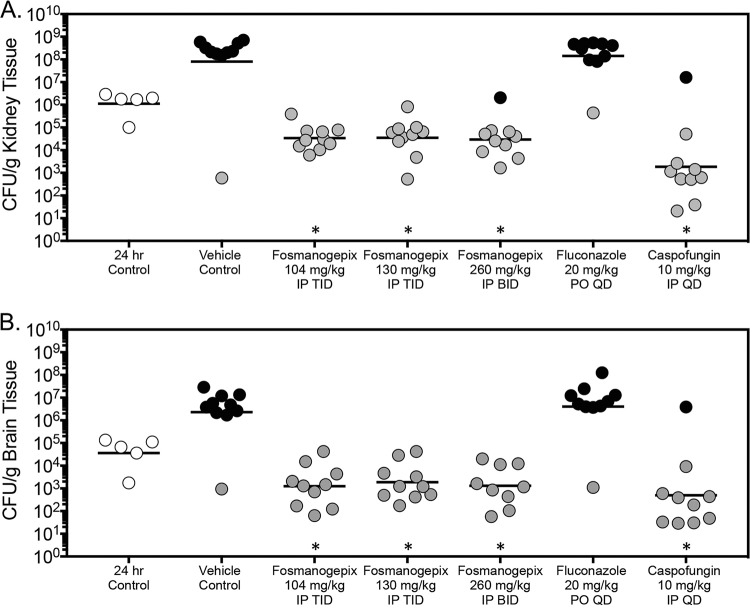
Kidney (A) and brain (B) fungal burden (CFU/g) in mice with invasive candidiasis secondary to C. auris in the survival arm. CFUs were measured on day 8 postinoculation after 7 days of therapy. *n* = 10 mice in the vehicle control and treatment groups; *, *P* < 0.05 versus control; *n* = 5 mice in the 24-hour control group. Black circles represent mice that succumbed to infection prior to day 21; gray circles represent mice that survived to the survival endpoint.

Fungal burden was also assessed in the survival arm as mice succumbed to infection or at the predetermined endpoint (day 21 postinoculation). Here, CFU counts within the kidneys of mice treated at each dose level of fosmanogepix (range, 4.47 to 4.55 log_10_ CFU/g) were significantly lower than that observed in the vehicle control group (7.90 log_10_ CFU/g; *P* < 0.0001 for all comparisons) (Fig. 3A). Similar to the day 8 fungal burden results and consistent with the survival curves, high dose caspofungin also lowered fungal burden within the kidneys in the survival arm. Significant reductions were also observed in brain tissue fungal burden in the survival arm with each dose of fosmanogepix and caspofungin (fosmanogepix range, 3.10 to 3.27 log_10_ CFU/g; caspofungin, 2.70 log_10_ CFU/g; *P* < 0.0001 for all comparisons). Interestingly, in contrast to the results of the fungal burden arm, each fosmanogepix dose level, as well as high dose caspofungin, resulted in a reduction in fungal burden within the brains and kidneys of greater than 1 log_10_ CFU/g compared with that measured just prior to the start of therapy. This reduction observed with fosmanogepix ranged between −1.50 and −1.57 log_10_ CFU/g in the kidneys and between −1.29 and −1.44 log_10_ CFU/g in the brains. Consistent with the survival results and the day 8 fungal burden results, no reductions in CFU counts were observed within the kidneys or brain tissue of mice treated with fluconazole.

## DISCUSSION

Candida auris is an emerging pathogen of great clinical significance. This yeast has rapidly spread globally, with numerous outbreaks of infections and colonization now having been reported in many countries (1–3). There are several characteristics of this fungal species which make it particularly challenging to deal with, including, but not limited to high associated mortality rate, a high rate of antifungal resistance (1, 2), and reported resistance to multiple classes of clinically available antifungal agents (1, 3, 10, 11). In addition, it is known to form biofilms which make clinical eradication, of either infection or colonization, and removal from environmental surfaces extremely challenging (36). Currently, echinocandins are recommended for initial therapy for patients with C. auris infections (https://www.cdc.gov/fungal/candida-auris/c-auris-treatment.html). However, given the limitations of IV-only therapy, the emergence of strains resistant to echinocandins, and limited brain penetration, there is a need for new therapeutic candidates. Several new antifungals currently under development have demonstrated promising activity, *in vitro* and/or *in vivo*, against C. auris. These include the echinocandin rezafungin (formerly CD101) and the triterpenoid ibrexafungerp (formerly SCY-078), both of which inhibit the production of 1,3-β-d-glucan within the fungal cell wall (36–40). In addition, the tetrazole VT-1598, which acts in a similar fashion to the triazoles by inhibiting the biosynthesis of ergosterol (41), has also demonstrated *in vitro* and *in vivo* activity against C. auris (42).

Manogepix, which has a novel mechanism of action different than that of the triazoles, echinocandins, and polyenes, has been shown to have broad *in vitro* activity against numerous pathogenic fungi, including *Candida*, *Cryptococcus*, *Aspergillus*, *Fusarium*, and *Scedosporium* species (15–19, 24). This *in vitro* activity has translated into *in vivo* efficacy of fosmanogepix in experimental models of candidiasis, cryptococcosis, coccidioidomycosis, aspergillosis, and scedosporiosis (20–22, 24, 28, 43). This therapeutic candidate has also previously been reported to have *in vitro* and *in vivo* activity against C. auris. Using a neutropenic model, Hager et al. reported that significant improvements in both *in vivo* outcome measures (survival and fungal burden) were achieved in mice infected with C. auris (27). These results are similar to the ones we report in the current study. The *in vitro* results reported by Hager et al. for manogepix against 16 C. auris isolates are also similar to those observed in the current study (MIC_90_, 0.03 μg/ml in both). Interestingly, fosmanogepix appeared to be just as effective when therapy was delayed by 24 hours, as done in our study, compared with 2 hours postinoculation, as performed in the previous study.

In another study which evaluated the pharmacokinetics and pharmacodynamics (PK-PD) of APX001 against *Candida* species conducted by Zhao et al., fosmanogepix was also efficacious at reducing kidney fungal burden when assessed at 4 days in a mouse model that utilized a cyclophosphamide/cortisone acetate regimen to render mice neutropenic (28). Here, maximum reductions in C. auris fungal burden from the starting inoculum measured just prior to the start of therapy (2 hours after inoculation) ranged between 0.21 log_10_ CFU/g to 1.02 log_10_ CFU/g (mean, −0.69 log_10_ CFU/g) and the PK-PD parameter most closely associated with efficacy was the area under the concentration-time curve for the free, unbound fraction of a drug (*f*AUC)/MIC ratio. In mouse PK-PD studies, a net stasis outcome (lack of change in burden over the treatment period) has been correlated with clinical efficacy in humans, especially for the echinocandins (44). In the current study, the doses utilized resulted in exposures at or below those previously associated with stasis. Zhao et al. reported the mean and median 24-hour total drug AUC (tAUC)/MIC values resulting in stasis, which were 7,336.4 and 5,864.2, respectively, for C. auris (28), whereas in this study, the 24-hour *t*AUC/MIC ranged between 1,130 to 3,520 in the three APX001 dose cohorts used to assess efficacy. However, fosmanogepix maintained efficacy, as is evident by both reductions in fungal burden and improvements in survival, especially at the 260-mg/kg BID dose where the 24-hour *t*AUC/MIC value was approximately half that reported for stasis in the previous PK-PD study (28).

Interestingly, we observed greater reductions in fungal burden in the survival arm of the current study, where mice received 7 days of therapy and were observed through day 21, versus the day 8 cohort. This may be due to the fact that a greater inoculum concentration was used to infect mice in the survival arm of this model than the fungal burden arm. Alternatively, the recovery of neutrophils during the 10- to 21-day postinoculation period following the administration of 5-fluorouracil may have contributed to greater reductions in fungal burden in the survival arm. Other studies that have evaluated APX001 in murine models of invasive candidiasis caused by C. auris did not measure fungal burden in a survival arm or did not assess survival (27, 28).

The pharmacokinetics of manogepix after administration of the prodrug fosmanogepix in mice are different than those observed in humans, as this agent has been shown to undergo rapid metabolism in outbred strains (e.g., CD-1 or ICR) with half-lives ranging between 0.9 to 2.75 hours (26, 28). In contrast, in humans the half-life has been reported to be ∼2.5 days in healthy volunteers in phase 1 clinical trials (30, 31). In order to slow the clearance and improve its overall exposure in mice, others have coadministered the cytochrome P450 inhibitor 1-aminobenzotriazole to inhibit the rapid metabolism of manogepix observed in murine models and allow for higher total daily exposures. This strategy has been successfully used in studies of invasive candidiasis caused by species other than C. auris, invasive aspergillosis, and invasive scedosporiosis (20, 22, 25, 26, 28). However, this was not done in the current study or in the others that evaluated the *in vivo* efficacy of fosmanogepix against invasive C. auris infection (27, 28).

Overall, the results of the current study are encouraging and confirm those that have previously reported good *in vivo* efficacy for fosmanogepix against C. auris infections. However, further studies are warranted to determine the exact role of this investigational agent against invasive infections caused by C. auris. This may include, but is not limited to, work to determine if the *in vivo* activity may be further enhanced with higher exposures. Overall, the results of the current study suggest that manogepix, administered as the prodrug fosmanogepix, may be a future option for the treatment of C. auris infections.

## MATERIALS AND METHODS

### Antifungals.

For *in vitro* testing, stock solutions and initial dilutions of manogepix (Amplyx Pharmaceuticals, San Diego, CA), fluconazole, and caspofungin (Sigma-Aldrich, St. Louis, MO) powders were made in dimethyl sulfoxide (DMSO), and further dilutions were then made in RPMI 1640 (0.165 M MOPS [pH 7.0] without bicarbonate). For the *in vivo* invasive candidiasis model, a stock solution of fosmanogepix, the prodrug of manogepix, was prepared using 0.21 M NaOH with further dilutions in sterile 5% wt/vol dextrose (D5W) solution. The clinical intravenous formulations of fluconazole and caspofungin were used for dosing in the *in vivo* model.

### Candida auris isolates.

For *in vitro* susceptibility testing, 10 C. auris isolates from the CDC FDA Antibiotic Resistance (AR) Bank (https://www.cdc.gov/drugresistance/resistance-bank/index.html) and 3 clinical isolates received and identified as C. auris through DNA sequence analysis of the internal transcribed spacer (ITS) ribosomal DNA (rDNA) region by the Fungus Testing Laboratory at the University of Texas Health Science Center were used (45–47). Prior to *in vitro* testing, all isolates were subcultured twice on Sabouraud dextrose agar (SDA). A clinical isolate of C. auris (UTHSCSA DI17-46) was used to establish the infection in mice. Prior to inoculation, the isolate was subcultured twice onto SDA, and colonies were taken from the second subculture and placed into brain heart infusion broth, which was grown overnight with shaking at 200 rpm at 37°C. Following centrifugation, the cells were collected and washed in sterile saline with 0.1% Tween 20. A hemocytometer was used to adjust the numbers of cells to the desired starting inocula, and viability was confirmed by serial dilution of aliquots onto SDA and counting the number of colonies following incubation at 37°C.

### *In vitro* susceptibility.

*In vitro* susceptibility testing was performed by broth microdilution in RPMI according the methods in the CLSI M27 standard (48). MICs were determined visually for manogepix, fluconazole, and caspofungin after 24 hours of incubation as the lowest drug that inhibited 50% growth. The concentration ranges evaluated were 0.002 to 1 μg/ml for manogepix, 0.125 to 64 μg/ml for fluconazole, and 0.015 to 8 μg/ml for caspofungin.

### Pharmacokinetics and dose tolerability.

A preliminary dose tolerability and pharmacokinetic study was done in uninfected, immunosuppressed mice. Two days prior to the start of dosing with the prodrug fosmanogepix, mice were rendered neutropenic with a single 5-mg intravenous (i.v.) dose of 5-fluorouracil. Antibiotic prophylaxis with enrofloxacin (50 ppm in the drinking water) was also provided to prevent bacterial infections. Fosmanogepix was administered by intraperitoneal (i.p.) injection at doses of 78 mg/kg TID, 130 mg/kg TID, and 260 mg/kg BID or TID. As a result of the 1.3-fold difference in the molecular weight for the prodrug versus the active moiety, these prodrug doses corresponded to manogepix doses of 60 mg/kg, 100 mg/kg, and 200 mg/kg, respectively. After the first dose on day 7 of therapy, groups of 3 mice per dose group were humanely euthanized at 0.5, 1, 2, 4, 8, 12, and 24 hours, and blood was collected by cardiac puncture. The plasma was separated and stored frozen. Plasma was shipped to QPS, LLC, and manogepix plasma concentrations were measured by an established liquid chromatography-tandem mass spectrometry (LC/MS-MS) assay in mouse plasma, with a lower limit of quantitation of 50.0 ng/ml. Pharmacokinetic parameters for manogepix were calculated using the noncompartmental method of the pharmacokinetic software package Phoenix WinNonlin (Certara, St. Louis, MO), and the area under the concentration-time curve (AUC) was calculated using the linear trapezoidal method.

### Infection model.

As noted above, mice were rendered neutropenic with a single 5-mg i.v. dose of 5-fluorouracil given 1 day prior to inoculation. This immunosuppression regimen results in neutrophil counts of <100 cells/mm^3^ for 10 days (49). Enrofloxacin was administered in the drinking water (50 ppm) to prevent bacterial superinfection. Mice were infected on day 0 with a 0.2-ml inoculum of the fluconazole-resistant strain C. auris (UTHSCSA DI17-46) via the lateral tail vein in order to establish infection. The target inocula were 1 × 10^7^ cells/mouse in the survival arm and 5 × 10^6^ cells/mouse in the fungal burden arm. Therapy began 24 hours postinoculation and was continued through day 7. Treatment groups consisted of vehicle control (D5W); fosmanogepix at doses of 104 mg/kg i.p. TID, 130 mg/kg i.p. TID, and 260 mg/kg i.p. BID; fluconazole at 20 mg/kg by oral (p.o.) gavage QD; and caspofungin at 10 mg/kg i.p. QD. The doses of fosmanogepix corresponded to doses of 80 mg/kg, 100 mg/kg, and 200 mg/kg of the active moiety manogepix. Mice were observed multiple times per day to minimize unnecessary pain and distress, and any animal that appeared moribund prior to the study endpoint was humanely euthanized. The animal protocol was approved by the University of Texas Health Science Center Institutional Animal Care and Use Committee.

### Fungal burden and survival.

Both fungal burden and survival arms were included. In the fungal burden arm, on day 8 postinoculation, mice were humanely euthanized, and the kidneys and brains were collected aseptically. The organs were homogenized, and further dilutions were made in sterile saline, which were plated onto SDA. Colonies were counted after 48 hours of incubation, and the number of CFUs per gram of tissue (CFU/g) were determined. In the survival arm, mice were followed for 14 days after therapy stopped until day 21 postinoculation. As noted previously, mice were monitored multiple times per day, and any animal that appeared moribund was humanely euthanized and death was recorded as occurring the next day. Fungal burden was also measured in the kidneys and brains on day 21 or as the mice became moribund as described above.

### Data analysis.

Descriptive statistics were used to evaluate the *in vitro* activity of manogepix, including MIC range, concentrations that inhibited 50% and 90% of the isolates tested (MIC_50_ and MIC_90_, respectively), and geometric mean (GM) MICs. Kaplan-Meier analysis was used to plot survival, and the differences in median survival and percent survival were evaluated by the log-rank and Fisher’s exact test, respectively. Analysis of variance (ANOVA) with Tukey’s posttest for multiple comparisons was used to assess for differences in fungal burden versus the vehicle control group, and *in vivo* fungicidal activity was defined as a 1-log_10_ CFU/g reduction in fungal burden compared with that of the 24-hour control group (i.e., fungal burden measured just prior to the start of therapy).
